# Surfactant-induced hole concentration enhancement for highly efficient perovskite light-emitting diodes

**DOI:** 10.1038/s41563-025-02123-y

**Published:** 2025-03-05

**Authors:** Jiajun Qin, Jia Zhang, Xianjie Liu, Yu Wang, Heyong Wang, Utkarsh Singh, Yanyan Wang, Haoliang Wang, Tianxiang Hu, Yiqiang Zhan, Yipeng Tang, Bin Hu, Constantin Bach, Carsten Deibel, Wei-Xin Ni, Sergei I. Simak, Igor A. Abrikosov, Mats Fahlman, Feng Gao

**Affiliations:** 1 Department of Physics, Chemistry and Biology, Linköping University, Linköping, Sweden; 2 The State Key Laboratory of Photovoltaic Science and Technology, Shanghai Frontiers Science Research Base of Intelligent Optoelectronics and Perception, Institute of Optoelectronics, Fudan University, Shanghai, P. R. China; 3 Laboratory of Organic Electronics, Department of Science and Technology, Linköping University, Norrköping, Sweden; 4 Center for Micro Nano Systems, School of Information Science and Technology, Fudan University, Shanghai, P. R. China; 5 Department of Materials Science and Engineering, University of Tennessee, Knoxville, TN USA; 6 Institut für Physik, Technische Universität Chemnitz, Chemnitz, Germany; 7 Department of Physics and Astronomy, Uppsala University, Uppsala, Sweden

**Keywords:** Electronic devices, Organic LEDs, Inorganic LEDs

## Abstract

It is widely acknowledged that constructing small injection barriers for balanced electron and hole injections is essential for light-emitting diodes (LEDs). However, in highly efficient LEDs based on metal halide perovskites, a seemingly large hole injection barrier is usually observed. Here we rationalize this high efficiency through a surfactant-induced effect where the hole concentration at the perovskite surface is enhanced to enable sufficient bimolecular recombination pathways with injected electrons. This effect originates from the additive engineering and is verified by a series of optical and electrical measurements. In addition, surfactant additives that induce an increased hole concentration also significantly improve the luminescence yield, an important parameter for the efficient operation of perovskite LEDs. Our results not only provide rational design rules to fabricate high-efficiency perovskite LEDs but also present new insights to benefit the design of other perovskite optoelectronic devices.

## Abstract (web summary)

Additives help to increase the surface hole concentration in metal halide perovskites, enabling high electroluminescence yields with low operating voltages.

## Main

The efficient operation of light-emitting diodes (LEDs) requires a bright emitter for radiative recombination as well as balanced charge injection with an equal number of injected electrons and holes into the emitter^1–4^. For conventional LEDs, the requirement of balanced charge injection can often be translated into relatively small injection barriers for both electrons and holes that enable operation at low bias voltages^5,6^.

During the past decade, metal halide perovskites have emerged as a promising emitter for a new generation of LEDs, owing to their unique features such as high defect tolerance^7,8^, high colour purity^9–11^ and easy tunability^12–14^. The external quantum efficiency (EQE) of state-of-the-art perovskite LEDs (PeLEDs) has been improved to high values over 30% (refs. ^15–20^). Very interestingly, in contrast to the small injection barrier requirements in conventional LEDs, a large hole injection barrier (ranging from 0.6 eV to 0.9 eV) is widely observed in highly efficient PeLEDs with low turn-on voltages (turn-on voltage here is defined as the corresponding threshold voltage for brightness exceeding 1 cd m^−^^2^)^9,15,21–24^. Moreover, the EQEs of the PeLEDs with large hole injection barriers were reported to be even higher than those with small injection barriers in some cases^24^. These abnormal phenomena indicate a new regime of mechanisms involved in the operation of PeLEDs and also new requirements in terms of device design strategies for PeLEDs.

In the present work, we construct a green-emitting PeLED with a seemingly large hole injection barrier. The hole injection layer is composed of an insulating polymer and another hole transport polymer, providing a situation that seems difficult for hole injection. Surprisingly, our device demonstrates a low turn-on voltage of only 2.0 V (which is distinctly lower than the optical bandgap of 2.39 eV of our perovskites) and a maximum EQE as high as 28.3% at a low current density of 0.025 mA cm^−2^. A range of optical and electrical measurements indicate increased hole concentration at our perovskite surface, where these holes are induced by the additives we use for device engineering. This increased hole concentration rationalizes the fact that turn-on voltages are dependent on electron-transport materials in both our and reported PeLEDs despite large hole injection barriers, where better electron-transport properties correspond to lower turn-on voltages. In addition, we find that increased hole concentration also contributes to enhance the device performance from another effect, which is to improve the luminescence yield (a factor equally important for efficient LEDs). Our results highlight the importance of surfactant-additive-induced hole concentration enhancement for high-efficiency PeLEDs, and we expect that the mechanisms can also be generalized to other LED technologies where similar carrier concentration enhancement (such as doping) plays a role.

## Characterizations of perovskite thin films

**Extended Data Fig. 1:**
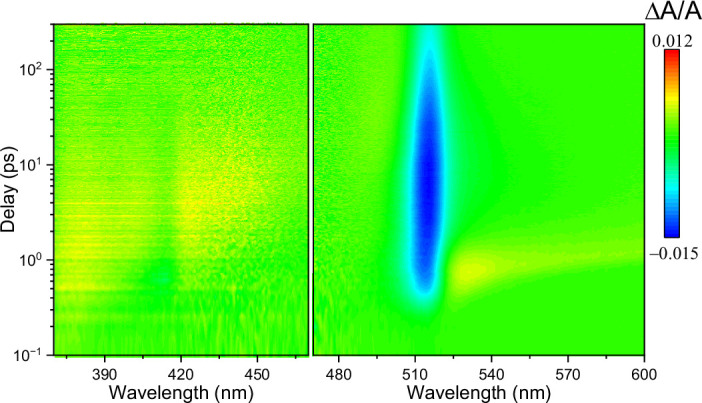
Transient absorption results of our perovskite thin film. Left: in UV range; right: in the visible range. Source data

Ruddlesden–Popper (RP) perovskites based on caesium lead bromide are used in our experiments owing to the easy one-step spin-coating process with good tunability. Specifically, the mixture of several precursors (*n*-butylammonium bromide (BABr), caesium bromide (CsBr), lead bromide (PbBr_2_) and additives) with a desired molar ratio enables the formation of mixed-dimensional <*n>* = 5 RP perovskites^25,26^ (details in Methods). The ultraviolet (UV)–visible absorption (Supplementary Fig. 1) shows only a small step-like absorption shoulder at ~410 nm and a steep absorption edge at ~520 nm. This step-like shoulder cannot be attributed to any low-*n*-value two-dimensional nanoplates because the absorption peaks featured in *n* = 1 and *n* = 2 nanoplates are located at ~403 nm and ~434 nm, respectively, with Gaussian-like shapes^27^. We attribute the shoulder at ~410 nm to the high-energy side absorption or excited-state absorption^28^. Transient absorption (TA) results with negligible photobleaching signal at wavelengths below 500 nm in Extended Data Fig. 1 further confirm the absence of *n* < 4 phases^27,29^. Thus, the radiative recombination of our perovskite films is channelled through free electron–hole bimolecular recombination, owing to small exciton binding energies that are typical for *n* > 4 RP perovskites and 3D perovskites^30^.

## PeLED performance

**Fig. 1:**
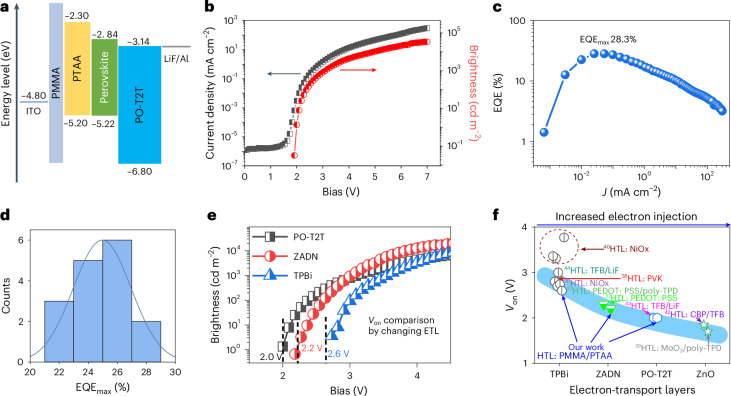
Device performance of the PeLED. **a**, A schematic energy diagram of the PeLED architecture. **b**,**c**, *J*–*V*–*L* curve (**b**) and EQE curve (**c**) of our champion PeLED. **d**, Statistical maximum EQE values of 16 PeLEDs. **e**, A comparison of turn-on voltages based on three different electron-transport layers (ETLs): PO-T2T, ZADN and TPBi. **f**, Our and published turn-on voltages of different green-colour-emitting PeLEDs using different electron-transport layers (the corresponding hole transport layers (HTLs) are listed). Source data

**Extended Data Fig. 2:**
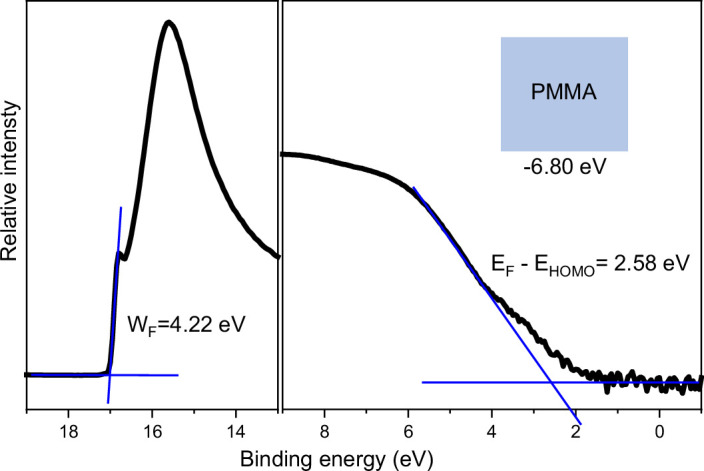
Energy level determination of PMMA. UPS result of PMMA to determine the HOMO level and the LUMO level, determined by combining HOMO level and the optical bandgap. Source data

**Extended Data Fig. 3:**
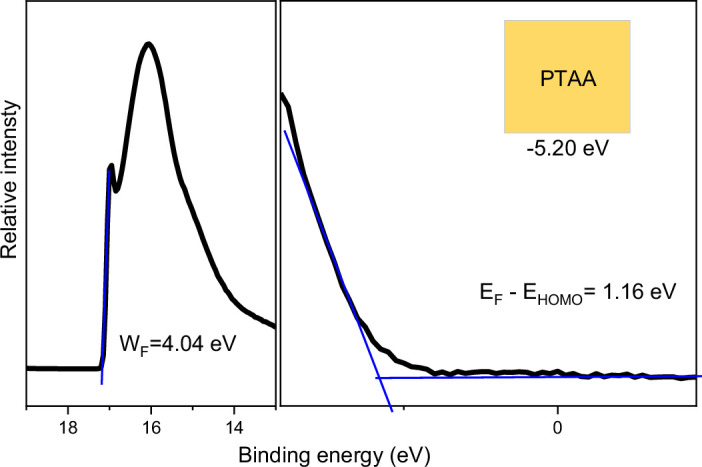
Energy level determination of PTAA. UPS result of PTAA to determine the HOMO level and the LUMO level, determined by combining HOMO level and the optical bandgap. Source data

**Extended Data Fig. 4:**
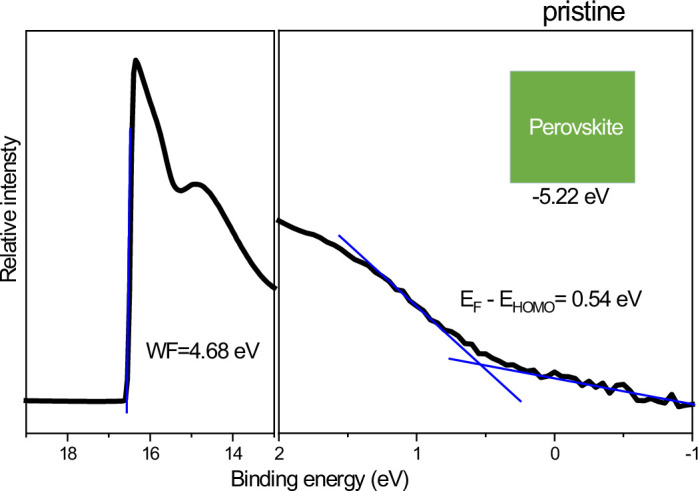
Energy level determination of our pristine perovskite thin film. UPS result of the pristine perovskite thin film to determine the HOMO level and the LUMO level, determined by combining HOMO level and the optical bandgap. Source data

We integrate the above-mentioned perovskite film into a PeLED based on the device architecture of indium tin oxide (ITO)/poly(methyl methacrylate) (PMMA)/poly[bis(4-phenyl)(2,4,6-trimethylphenyl)amine] (PTAA)/perovskite/2,4,6-tris[3-(diphenylphosphinyl)phenyl]-1,3,5-triazine (PO-T2T)/lithium fluoride (LiF)/aluminium (Al) (Supplementary Fig. 2). The energy diagram of the device in Fig. 1a is determined by combining UV photoelectron spectroscopy (UPS) results (Extended Data Figs. 2–4), the optical bandgap of the perovskite film (Supplementary Fig. 3) and the literature results^31,32^. A thin (~3 nm) insulating PMMA layer is inserted in between anodic ITO and PTAA, resulting in a seemingly large energy barrier for hole injection from the anode to the perovskite. The current density (*J*)–bias voltage (*V*) curves of the hole-only device with the structure of ITO/PMMA/PTAA/perovskite/poly (9,9-dioctylfluorene-alt-*N*-(4-sec-butylphenyl)-diphenylamine) (TFB)/molybdenum (VI) oxide (MoO_3_)/silver (Ag) in Supplementary Fig. 4 further shows that the hole injection is not efficient (with current densities even lower than 1 mA cm^−2^ at 3 V). On the cathode side of the LED structure, the energetic levels indicate a relatively small barrier for the electron injection from Al to the perovskite.

For conventional LEDs, such a device structure with a large hole injection barrier and a small electron-injection barrier often leads to a large turn-on voltage of the device due to low injection current densities and, thus, low EQE at low biases^33,34^. However, in the current case, the device shows excellent electroluminescence (EL) performance at low working biases. As shown in the current density–voltage–brightness (*J*–*V*–*L*) data in Fig. 1b and the EQE curve in Fig. 1c, the device turn-on voltage is as low as 2.0 V, and the maximum brightness and EQE are as high as 33,596 cd m^−2^ and 28.3%, respectively. The EL spectral peak remains nearly unchanged over a wide range of biases (Supplementary Fig. 5), with the peak position fixed at 518 nm. The statistical maximum EQE values of 16 pixels are included in Fig. 1d, with an average value of 25.0%. Noticeably, the maximum EQE occurs at a low current density of 0.025 mA cm^−2^ (under the bias of 2.2 V). In such a low current density, we can still achieve rather high EQE, implying that the internal quantum efficiency of the perovskites is high at low carrier densities. We should point out that, even in the case with a high hole injection barrier, the holes can still be injected at relatively low bias. As shown in Fig. 1a, the energy diagram indicates a built-in field of ~1.66 V at zero bias. This means that, once the bias voltage exceeds 1.66 V, there is the possibility of injecting holes through Fowler–Nordheim tunnelling and the injection ability increases significantly with increasing bias, as indicated by the *J*–*V* curve in Fig. 1b. Thus, for our case with the turn-on voltage of ~2 V and maximal EQE point at ~2.2 V, there is hole replenishment to maintain the high EQE.

We then substitute the electron-transport layer PO-T2T with 2-[4-(9,10-di-naphthalen-2-yl-anthracen-2-yl)-phenyl]-1-phenyl-1*H*-benzoimidazole (ZADN) or 2,2′,2′′-(1,3,5-benzinetriyl)-tris(1-phenyl-1-*H*-benzimidazole) (TPBi). As compared with PO-T2T, ZADN and TPBi have relatively lower electron mobility^35,36^, suggesting decreased electron-injection ability in the ITO/PMMA/PTAA/perovskite/TPBi or ZADN/LiF/Al devices. By comparing the brightness versus bias voltage curves of these three devices in Fig. 1e, we find that the device turn-on voltage increases from 2.0 V to 2.2 V and 2.6 V upon the substitution of PO-T2T with ZADN and TPBi, respectively.

**Extended Data Fig. 5:**
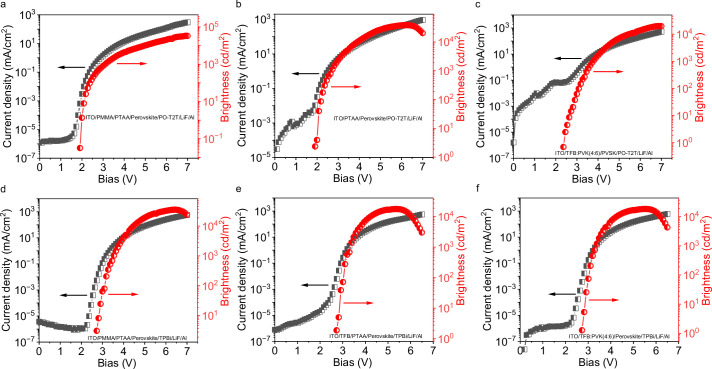
J-V-L curves of different PeLEDs. **a**, ITO/PMMA/PTAA/Perovskite/PO-T2T/LiF/Al, **b**, ITO/PTAA/Perovskite/PO-T2T/LiF/Al, **c**, ITO/TFB:PVK(4:6)/Perovskite/PO-T2T/LiF/Al, for PO-T2T based devices and **d**, ITO/PMMA/PTAA/Perovskite/TPBi/LiF/Al, **e**, ITO/TFB/PTAA/Perovskite/TPBi/LiF/Al, **f**. ITO/TFB:PVK(4:6)/Perovskite/TPBi/LiF/Al, for TPBi based devices. Source data

Interestingly, after collecting the literature results^23,37–44^ on how the transport layer affects the turn-on voltage in other green-colour-emitting PeLEDs, we observe a similar phenomenon that the turn-on voltages are mainly limited by the electron-transport layers. As shown in Fig. 1f, the electron-injection ability increases from TPBi to ZADN and PO-T2T and then to zinc oxide, owing to the increased electron mobility. The minimum turn-on voltage is decreased from 2.6 V to 2.3 V, 2.0 V and 1.7 V, respectively. The decrease of the turn-on voltages matches well with the increase of electron mobility, despite different hole transport layers used in different PeLEDs (Supplementary Table 1). A similar phenomenon has also been verified in our PeLEDs, where the minimal achievable turn-on voltage is determined by the electron-transport layer despite different hole transport layers used (Extended Data Fig. 5). This trend implies that the turn-on voltage might be further reduced if the electron injection is further facilitated.

## Additive-induced hole concentration enhancement

To rationalize turn-on voltages determined by the electron injection and efficient operation of PeLEDs at low current densities, we propose that a high hole concentration exists at the perovskite surface. On the one hand, it makes electron injection more difficult, which balances the relatively poor hole injection. On the other hand, it increases the radiative recombination channels by providing excess holes. Typically, the hole concentration enhancement in semiconductors can be caused by p-type doping or the charge transfer process with similar effects, where dopants are expected to withdraw electrons from the valence band (VB). The dopants can be in the bulk of perovskites or at the perovskite boundaries^45,46^. We exclude the possibility of bulk dopants because of absence of the effective A-site or B-site substitutional elements in our precursor solutions. Therefore, we tend to believe that doping or charge transfer occurs at the perovskite grain boundaries, where additives wrap the perovskite grains. Typically, the effect of the additives is to passivate the perovskite grain boundaries by bonding with the uncoordinated dangling bonds of Pb and/or improving the crystallinity during the perovskite crystallization process^47^. Here, we hypothesize that the additives might also play a role in increasing the hole concentration at the perovskite surface.

**Fig. 2:**
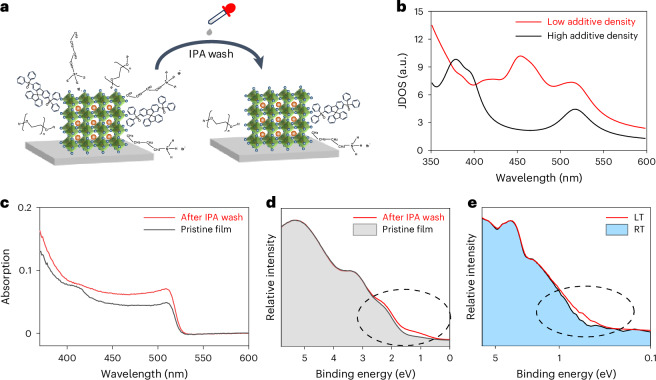
Hole concentration enhancement in the perovskite film through additive engineering. **a**, A schematic diagram of controlling the amount of additives by the IPA washing method. **b**, The calculated JDOS of these configurations with different additive densities to illustrate the corresponding change in absorption characteristics after IPA wash. **c**,**d**, Absorption (**c**) and UPS spectra (**d**) of the pristine film and the film after IPA washing at room temperature. **e**, UPS spectra of the pristine film at room temperature (RT) and low temperature (LT) (~80 K). Source data

**Extended Data Fig. 6:**
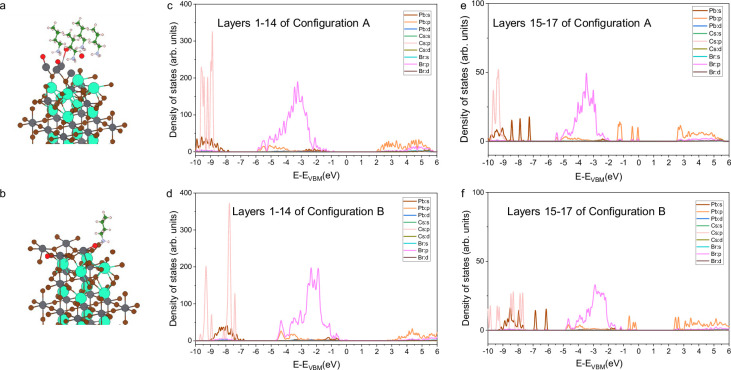
Electronic structure calculations at the level of Density functional theory with the HSE06 functional. **a**, Configuration A: Illustration of model structure with four adsorbed BABr additives **b**, Configuration B: Illustration of model structure with one adsorbed BABr additive. DOS (density of states) of **c, d** (layers 1-14, corresponding to the bulk) and **e, f** (layers 15-17, corresponding to the surface) calculated for configuration A (**c**,**e**) and configuration B (**d**,**f**). Source data

**Extended Data Fig. 7:**
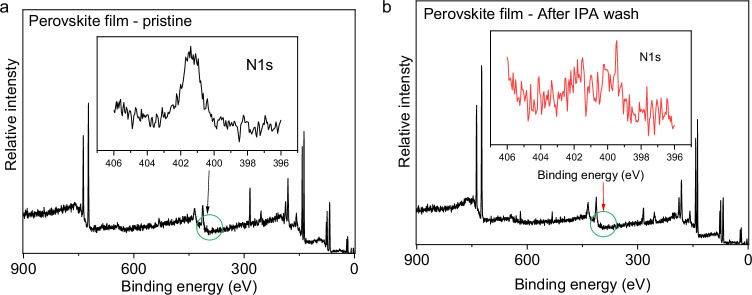
X-ray photoelectron spectroscopy (XPS) results of our perovskite films. **a**, the pristine perovskite film. **b**, the perovskite film after IPA wash. Source data

To verify our hypothesis, we perform electronic structure calculations at the level of density functional theory (DFT) for two perovskite model configurations: configurations A and B. Configuration A (Extended Data Fig. 6a) involves a perovskite slab consisting of 17 alternating Pb–Br_2_ and Cs–Br layers. Four BABr molecules are adsorbed on the top layer with Pb–Br termination. Configuration B (Extended Data Fig. 6b) involves the same perovskite slabs but with the surface density of additives reduced to one-fourth of configuration A. These two situations with more and fewer surface additives can be revealed by our post-treatment experiments, where the perovskite thin film is washed by 2-propanol (IPA) (as illustrated in Fig. 2a). Because the perovskite grains are insolvable in IPA, but excess additives such as BABr are solvable, the IPA washing process leads to the removal of most BABr additives (indicated by the X-ray photoelectron spectroscopy results in Extended Data Fig. 7), corresponding to the change from configuration A to configuration B with higher and lower surface density of BABr additives, respectively.

As shown in Extended Data Fig. 6c,d, the projected density of states (DOS) for layers 1–14 remains nearly unchanged for both configurations. However, notable changes are observed in the DOS corresponding to top layers 15–17 (Extended Data Fig. 6e,f), which lie closer to the adsorbed additives. These results suggest that the adsorbed additives at the perovskite surface affect the surface electronic structure, providing the possibility to increase the surface hole concentration. Specifically, in configuration A with an increased number of adsorbates, there are fewer available states in the DOS near the VB maxima (VBM) than in configuration B, suggesting a reduced number of electronic states near the VBM.

This decrease in the number of accessible states near the VBM can significantly influence the joint DOS (JDOS). As shown in Fig. 2b, the calculated JDOS for configuration A (high additive density) is lower than that for configuration B (low additive density) near the optical bandgap of the perovskites (~520 nm), owing to the reduced number of states near the VBM. The variation in electronic states near the VBM suggests that the interaction between the additives and the perovskite surface may significantly influence the electronic structure. The mechanism behind this phenomenon could be related to electron-withdrawal properties of the additive adsorbate. For semiconductors, the absorption coefficient is directly proportional to the JDOS; thus, a lower JDOS corresponds to lower absorption. Our calculations, as shown in Fig. 2b, reveal that the absorption for configuration A is lower than that of configuration B near the optical bandgap of the perovskites (~520 nm).

The absorption spectral comparison in Fig. 2c indicates lower absorption for the pristine film (with more additives) than that of the IPA-washed film (with fewer additives), consistent with our JDOS results in Fig. 2b. Notably, this absorption reduction can also be reversed by adding the BABr additives back to the IPA-washed perovskite film (Supplementary Fig. 6), further indicating that the insertion of BABr additives induces the surface effect on perovskites. It should be mentioned that this additive-induced absorption reduction resembles the classical p-type doping effect where VBM electrons are withdrawn. With fewer accessible states, the number of possible optical transitions decreases, leading to reduced absorption in the doped system^48,49^.

**Extended Data Fig. 8:**
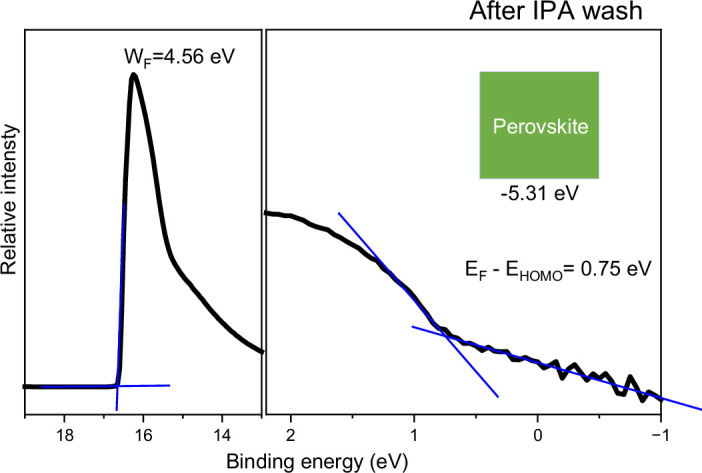
Energy level determination of perovskite thin film after IPA wash. UPS result of the perovskite thin film after IPA wash to determine the HOMO level and the LUMO level, determined by combining HOMO level and the optical bandgap. Source data

To further demonstrate the electron-withdrawing effect at the VBM, we compare the UPS results of these two perovskite thin films, as shown in Fig. 2d. The UPS intensity in the low-binding-energy region (corresponding to the DOS near the VBM) for the pristine film is lower than that of the IPA-washed film, indicating that the number of states near the VBM is reduced with more additives at the perovskite surface. This confirms that charge transfer occurs where electrons are indeed withdrawn from the perovskite surface. This charge transfer process is also confirmed by the work function (*W*_F_) change from 4.68 eV (see Extended Data Fig. 4 for the pristine perovskite film with more additives) to 4.56 eV (see Extended Data Fig. 8 for the IPA-washed film with fewer additives). With electrons being withdrawn from the surface by the additives (the pristine film), *W*_F_ increases. Such an increase is further indicated by the conducting atomic force microscopy results in Supplementary Fig. 7. The current is always higher in perovskite grains than the surroundings in two perovskite systems. By averaging the current in the same selected zone area, the current for the pristine film is higher than the film after IPA wash, where the current of the perovskite films is determined by both hole injection and hole transport. Considering that the bulk hole concentrations of these two perovskite films are low, the current difference is determined mainly by the hole injection barrier. With more additives on top in the pristine film, *W*_F_ is higher, matching well with the increased current. The *W*_F_ shift is also an indication that charge transfer occurs at the perovskite interface by adding additives (in the pristine film), which enable the enhancement of hole concentration at the perovskite surface.

Figure 2e shows the increased UPS intensities at low temperatures (~80 K), indicating that this charge transfer process is suppressed. Thus, we conclude that the hole concentration enhancement is a thermally activated process. This surface effect is also like the conventional dedoping process where the doping concentration is reduced at low temperatures. We then rationalize this charge transfer process by considering the physical properties of the thermal activation. The incorporation of additives has been demonstrated to effectively coordinate with dangling bonds and passivate the deep trap states at the perovskite grain boundaries; at the same time, the coordination between perovskite with the additives also results in crystal imperfections, leading to the formation of shallow defects^50^. The thermally activated hopping process occurs dynamically to trap electrons from the VBM, leaving holes in the VBM and resulting in a hole concentration enhancement.

## Hole concentration enhancement improving the luminescence yield

**Fig. 3:**
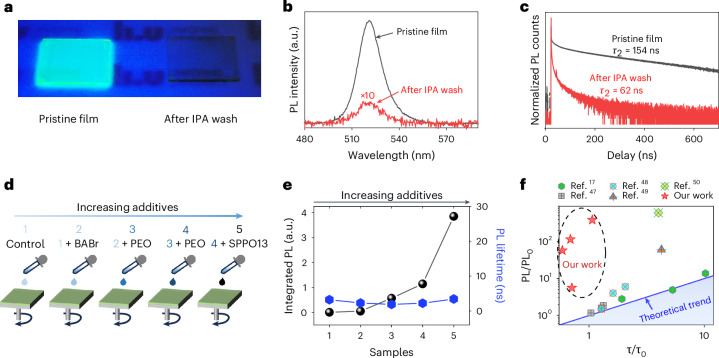
Additive-induced hole concentration improving the perovskite luminescence yield. **a**, A photo of two perovskite films (the pristine film with a large amount of additives and IPA-washed film with a lower amount of additives) under UV light illumination. **b**,**c**, PL spectra (**b**) and normalized PL lifetime (**c**) of these two perovskite films based on the same measurement setups. **d**, A schematic diagram to control the amount of additives through changing the precursor compositions, with the amount of additives continuously increasing from sample 1 to sample 5. **e**, PL lifetimes and absolute PL intensities of these five perovskite samples. **f**, Our and published results where the PL intensity enhancement and PL lifetime increment are compared. The solid blue line is the theoretical prediction based on the defect passivation model. Source data

**Extended Data Fig. 9:**
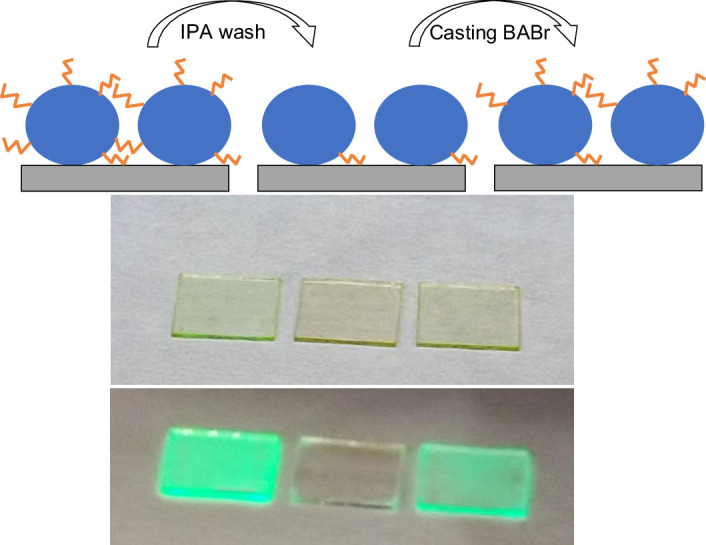
Post-treatment of our perovskite film with sequential IPA washing and BABr addition. The upper row shows the schematic diagram of our treatment. The middle row and bottom row show the corresponding photos under ambient light and UV light illumination.

Now we explore the hole concentration enhancement effect on the luminescence yield of our perovskites, another equally important factor responsible for efficient operation at low current densities. Figure 3a indicates that decreasing the quantity of additives via IPA wash leads to significantly decreased luminescence yield, shown by the photograph of perovskite films under UV lamp illumination. The photoluminescence (PL) spectra in Fig. 3b further show that the PL intensity of the optimized pristine film (higher hole concentration by adding more additives) is 48 times higher than that of the IPA-washed film (lower hole concentration with fewer additives). Such a PL intensity change is directly related to the incorporation of additives, visualized by the photograph of the perovskite film with removal or addition of BABr in Extended Data Fig. 9.

Because the PL peak position remains at the same energy (Fig. 3b) after IPA wash, the nature of the radiative recombination (that is, free carrier recombination as explained previously) is not changed. It might be tempting to attribute the PL enhancement to the additive-induced passivation. However, we exclude the possibility that PL enhancement here is caused mainly by additive-induced defect passivation through the comparison of the PL enhancement and PL lifetime enhancement. As shown in Fig. 3c, the PL lifetimes of the pristine film (with higher hole concentration induced by more additives) and the IPA-washed film (with lower hole concentration induced by fewer additives) are 154 ns and 62 ns, respectively. Thus, as compared with the IPA-washed film, PL lifetime enhancement of the pristine film is only 2.48 times while the PL intensity enhancement is over 48 times. This large deviation is not consistent with the defect passivation mechanism (that is, decreasing non-radiative recombination rate *Γ*_nr_) where the multiple of luminescence yield enhancement and the multiple of PL lifetime increment is supposed to be the same according to the ABC model^51^ (the detailed deduction can also be found in Supplementary Note 1).

The luminescence yield is proportional to the ratio between the radiative recombination rate (*Γ*_r_) and the total recombination rate (radiative (*Γ*_r_) + non-radiative (*Γ*_nr_)). Since we have now excluded the main contribution from the additive-induced passivation (by decreasing *Γ*_nr_), we believe that increasing *Γ*_r_ by hole concentration enhancement would be the main reason for the significant PL enhancement in our case. For materials with excess hole concentration of *p*_D_, the radiative recombination term in the case of free carrier recombination will be *Γ*_r_ = *bn*(*n* + *p*_D_), where *b* is the bimolecular recombination coefficient and *n* is the photogenerated carrier density. Hence, the quantum yield (QY) is expressed as

$${{\mathrm{QY}}}=\frac{{{\varGamma }}_{{\rm{r}}}}{{{\varGamma }}_{{\rm{r}}}+{{\varGamma }}_{{\rm{nr}}}}=\frac{{bn}(n+{p}_{{\mathrm{D}}})}{{an}+{bn}(n+{p}_{{\mathrm{D}}})},$$

where *a* is the trap-assisted non-radiative recombination constant. We can see that increasing hole concentration will significantly increase QY. With a small photogenerated carrier density *n* and ${p}_{{\mathrm{D}}}\gg n$, we then have *Γ*_r_ ≈ *bnp*_D_. This indicates that the logarithmic slope of PL intensity (proportional to *Γ*_r_) versus photoexcitation intensity (proportional to *n*)^51^ curve switches to 1 as compared with the factor of 2 in the perovskite film without hole concentration enhancement.

**Fig. 4:**
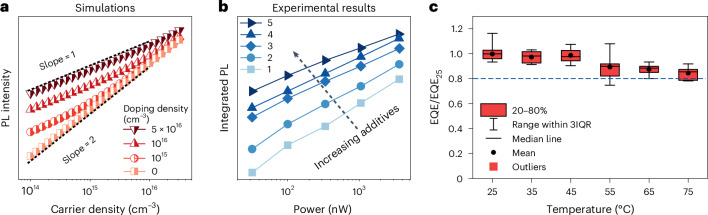
The effect of hole concentration enhancement on the power-dependent PL intensity and temperature-dependent EQE. **a**, Simulated carrier density dependent PL intensities at different excess hole concentrations. **b**, Power-dependent PL results of our five perovskite samples with different amounts of additives. **c**, Statistical maximal EQE results of 30 device batches at different temperatures ranging from 25 °C to 75 °C (note: two pixels of each device are tested, with one randomly selected for 25 °C and the other one randomly selected for a high-temperature measurement). IQR, interquartile range. EQE_25_, the average maximal EQE value measured at room temperature. Source data

**Extended Data Fig. 10:**
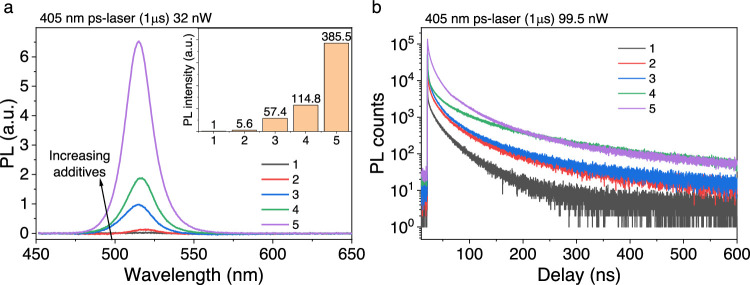
PL and PL lifetime of perovskite films with different amounts of additives. **a**, PL spectra and **b**, PL lifetimes of our 5 different perovskite samples with different amounts of additives, where the amount continuously increases from sample 1 to sample 5. Source data

We now design additional experiments to further confirm the luminescence yield enhanced by increased hole concentration. We sequentially increase the hole concentration by increasing the number of commonly used additives via adding BABr, poly(ethylene oxide) (PEO) and 2,7-bis(diphenylphosphoryl)-9,9′-spirobifluorene (SPPO13) in the precursor solutions and prepare the perovskite thin films (marked as 1, 2, 3, 4 and 5) correspondingly (Fig. 3d). Among these five samples, film 1 contains the lowest quantity of additives and film 5 contains the most, which corresponds to hole concentration enhancement from 1 to 5. By increasing the quantity of additives, PL intensity increases accordingly, with the maximum increment exceeding two orders of magnitude (Extended Data Fig. 10a). In contrast to the significantly improved PL intensity, the changes on the PL lifetime (Extended Data Fig. 10b) are small, ranging between 2 ns and 3.5 ns. This unmatched PL intensity enhancement and PL lifetime change (Fig. 3e) is, again, inconsistent with the conventional defect passivation mechanism based on the ABC model. Instead, it is consistent with the hole concentration enhancement-induced PL enhancement. For sample 5 with a large amount of additives, we observe a slope of 1 in the light-intensity-dependent PL plot (Fig. 4b), agreeing with our simulation results that take hole concentration enhancement into consideration (Fig. 4a, with the simulation details in Supplementary Note 2).

Similar surfactant-induced hole concentration enhancement phenomena have also been observed in other perovskite systems: the benchmark three-dimensional (3D) methylammonium lead bromide (MAPbBr_3_) (Supplementary Fig. 8) and 3D methylammonium lead iodide (MAPbI_3_) (Supplementary Fig. 9), where we combine UPS and power-dependent PL intensity measurements. As such, we believe that the effect of additive-induced surface hole concentration enhancement on the PL intensity is not limited to our materials; instead, it might be generally applicable to many perovskites, although not discussed previously. Indeed, when we summarize the literature results on PL intensity and PL lifetime, we notice a similar inconsistency between these two parameters in other perovskite systems (Fig. 3f), where the PL intensity enhancement sometimes goes far beyond the enhancement of PL lifetime^17,52–55^.

## Excellent EL performance at elevated high temperatures

Since hole concentration enhancement originates from the thermally activated process (Fig. 2e), we expect it to be maintained at high temperatures. We then characterize the EQE of the PeLED at elevated temperatures. As shown by the temperature-dependent normalized maximum EQE results in Fig. 4c, the maximum EQE value at 75 °C can still maintain 80% of its initial value at 25 °C. Such a minor drop at high temperature is rationalized when the defect-assisted *Γ*_r_ increment is still far below the hole concentration enhancement-induced high *Γ*_r_ at high temperatures. As such, we demonstrate the promise of our high-efficiency PeLEDs in lighting and display due to inevitably elevated operational temperatures by heat generation.

## Outlook

For a conventional LED, both electron injection and hole injection should be considered simultaneously to enable balanced injection for radiative recombination at low operational bias. By contrast, in the hole-concentration-enhanced LEDs, the devices can still work efficiently under low current densities despite high hole injection barriers, resulting in low turn-on voltages. We should point out that such a mechanism based on hole concentration enhancement has also been observed in the inorganic LEDs where doped emitters usually lead to excellent luminescence performance^56,57^.

In theory, the free electron–hole recombination allows identical phenomena for hole-rich (in our work) and electron-rich situations. Thus, in principle the device design discussed in this work can also be applied to the devices with high electron-injection barriers. In reality, most additive engineering we use here leads to hole concentration enhancement rather than electron concentration enhancement, as indicated by our experimental and DFT simulation results (Fig. 2). Similar hole concentration enhancement has also been reported in a very recent report^58^, where the authors introduced phosphonic acid molecular in the perovskite film and obtained high-performance PeLEDs.

In conclusion, we rationalize and demonstrate high-performance PeLEDs under a seemingly large hole injection barrier through additive-induced hole concentration enhancement at the perovskite surface. On the one hand, it provides extra barriers for electron injection, leading to turn-on voltages determined by the electron-transport layer; on the other hand, it increases the radiative recombination rate for significantly enhanced luminescence yield even at very low operational current densities. Our results provide rational guides towards high-performance PeLEDs with both high EQEs and low turn-on voltages. In addition, since additive engineering has been widely used for different perovskite optoelectronic devices, our understanding of the additive-induced surface effect can also provide new insights into the development of other perovskite optoelectronics.

## Methods

### Chemicals

The chemicals used were PMMA (from Sigma-Aldrich), PTAA (Mn, the number average molecular weight, 3,000–6,000, from Xi’an Polymer), BABr (from Sigma-Aldrich), PbBr_2_ (from Sigma-Aldrich), CsBr (from Sigma-Aldrich), PEO (Mw, the weight average molecular weight, 600k, from Sigma-Aldrich), guanidinium bromide (GABr, from Sigma-Aldrich), SPPO13 (from Sigma-Aldrich), ethanolamine (EA, from Sigma-Aldrich), l-phenylalanine (L-P, from Sigma-Aldrich), lead acetate trihydrate (Pb(Ac)_2_·3H_2_O, from Sigma-Aldrich), methylammonium bromide (MABr, from Greatcell Solar Materials), TPBi (from Lumtec), PO-T2T (from Lumtec), LiF (from Sigma-Aldrich), aluminium (Al, from Kurt J. Lesker), chlorobenzene (CB, from Sigma-Aldrich), IPA (from Sigma-Aldrich), dimethyl sulfoxide (DMSO, from Sigma-Aldrich) and *N*,*N*-dimethylformamide (from Sigma-Aldrich). All chemicals were used without further purification.

### Perovskite film preparation

For the preparation of R–P perovskite films 1–5, BABr, PbBr_2_, CsBr and PEO with the desired molar ratio (see Supplementary Table 2 for the detailed combinations) were dissolved in DMSO with a Pb concentration of 0.2 M to obtain solution A. Solution A was heated at 60 °C for a whole night to thoroughly dissolve all the precursors. Then, we mixed 200 µl of solution A with either 200 µl of DMSO (for films 1–4) or 200 µl of SPPO13 solution (8 mg ml^−1^ in DMSO) (for film 5) and 1 µl of EA to obtain the final solution for spin coating.

For the preparation of optimized R–P perovskite film for characterization and device fabrication, we partially replaced BABr with GABr. Specifically, solution B was prepared by mixing GABr (0.0336 g), PbBr_2_ (0.2202 g), CsBr (0.1277 g) and PEO (0.0075 g) in 3 ml DMSO. Then, 180 µl of solution A (the recipe used for films 4 and 5) was mixed with 35 µl of solution B, 200 ml of SPPO13 solution (8 mg ml^−1^ in DMSO) and 1 µl of EA to obtain the final solution for spin coating.

After dropping 50 µl of the final solution onto the ITO/PMMA/PTAA/L-P substrate, we spin coated the substrate at a rate of 5,000 rpm for 50 s. Finally, the substrate was put on a hotplate for 10 min with an annealing temperature of 80 °C.

For the preparation of MAPbBr_3_, 1 M Pb(Ac)_2_·3H_2_O and 3 M MABr were mixed in *N*,*N*-dimethylformamide (for films with the additive, 0.2 M BABr was added). Then, the clear solution was dropped on ITO substrate for spin coating (3,000 rpm for 60 s), followed by annealing at 60 °C for 25 min.

### Device fabrication

The ITO substrate was cleaned by ultrasonic treatment with detergent, deionized water (twice) and ethanol sequentially, with each cycle lasting at least 10 min. Then, the substrate was blown dry by nitrogen gas and exposed to UV ozone for at least 15 min. After that, the substrate was transferred into a nitrogen glovebox for film deposition.

First, PMMA solution (3 mg ml^−1^ in CB) was spin coated onto the ITO substrate at a rate of 3,000 rpm for 30 s, followed by annealing at 150 °C for 10 min. Second, PTAA solution (3 mg ml^−1^ in CB) was spin coated at a rate of 3,000 rpm for 30 s, followed by annealing at 120 °C for 10 min. Third, L-P solution (0.75 mg ml^−1^ in DMSO) was spin coated at a rate of 5,000 rpm for 60 s, followed by annealing at 80 °C for 10 min. Fourth, the perovskite solution was spin coated, as introduced in the last part. Fifth, the substrate was transferred into an evaporator for thermal evaporation. PO-T2T (48 nm, at a rate of 0.3 nm s^−1^), LiF (1 nm, at a rate of 0.01 nm s^−1^), Al (100 nm, at a rate of >0.5 nm s^−1^) was deposited sequentially.

### Characterizations and measurements

PL spectra of all the perovskite films were detected by using a 405 ps pulsed laser (EPL-405, from Edinburgh) with a focal lens (focal length, *f* = 50 mm), before a neutral density filter (NDC-100C-4, from Thorlabs). The emitted light signals were collected through a confocal lens (Mitutoyo Apochromatic Objectives, MY10X-803, from Thorlabs) with fibre (M124L02, from Thorlabs), directing to the spectrometer (a spectrograph (Shamrock 303i) combined with a detector (Andor Newton DU970N-UVB CCD-6198 (Si)), from Andor).

Time-correlated single-photon counting measurements were also performed with the 405 ps pulsed laser as the excitation. The emission signal went through a long-pass filter (FELH0450, from Thorlabs), followed by the confocal lens and the fibre that directed to the single-photon detector (id100, from IDQ) and a time-to-digital converter (quTAG standard, from qutools). Absorption spectra were measured with a PerkinElmer model Lambda 900. UPS measurements were carried out in ultrahigh-vacuum surface analysis system equipped with a Scienta ESCA200 hemispherical analyser and a standard He-discharge lamp He I (21.22 eV). The spectrometer was calibrated with the reference of Fermi level (0.0 eV). The total energy resolution of UPS was about 0.08 eV, as estimated from the width of the Fermi level. A negative bias of −3 V was applied to the sample during UPS measurement to extract its work function. All spectra were recorded at normal emission and room temperature. For low-temperature operation, liquid nitrogen was used to cool down the temperature of the sample.

The PeLED performance was characterized with a home-built testing platform in the nitrogen glovebox. Specifically, the devices were placed on top of the integrating sphere, with the ITO glass side downwards. Two probe stations were connected to the device anode (ITO) and cathode (Al) for the *J*–*V* scanning by Keithley 2400 source meter. Simultaneously, the EL signal was collected by a QE65 Pro spectrometer through a fibre connected to the integrating sphere. The active device area of the LEDs was 0.0725 cm^2^. The *J*–*V*–L scanning is from 0 V to 7 V, with the homogeneously distributed total scanning points of 78. The number of power line cycles is 0.1, and the delay between two points is 100 ms. For the temperature-dependent EL measurement, a hotplate was placed next to the heating surface toward the integrating sphere. During the measurement, a thermometer was used to record the temperature. We fabricated 30 devices, with each device containing six pixels. Two pixels of each device were characterized, with one pixel measured at room temperature (25 °C) and the other was used for high-temperature measurements.

### Electronic structure calculations

#### Calculation parameters

All DFT calculations were performed in the framework of the projector augmented wave^59^ method, as implemented in the Vienna ab initio simulation package^60–62^. A cut-off energy of 450 eV was used for the plane wave expansion of the Kohn–Sham orbitals. The exchange-correlation energy was approximated using the PBEsol^63^ and hybrid HSE06^64^ functionals, including spin–orbit coupling.

The two configurations, A (Extended Data Fig. 6a) and B (Extended Data Fig. 6b), were modelled as 17 alternating Cs–Br and Pb–Br_2_ layer slabs with four and one adsorbed additive molecules, respectively.

The lattice constant was fixed at the relaxed PBEsol equilibrium value. While ionic positions of layers 1–14 were fixed, layers 15–17 were relaxed with the HSE06 functional with a *k*-point density of 0.30 Å^−1^ (where **k** is the reciprocal space wave vector) and a Gaussian smearing with a width of 0.05 eV until the residual forces <5 × 10^−2^ eV Å^−1^ were achieved. Both PBEsol and HSE06 functionals underestimate the bandgap of this material; hence, a scissor shift is utilized to match the experimentally observed bandgap. A value of *σ* = 0.10 is used for the calculation of JDOS in Fig. 2b.

#### JDOS

For a semiconductor, the optical absorption in direct band-to-band transitions is proportional to^65^

1 $$\frac{2\uppi }{{{\hslash }}}{\int }_{\text{BZ}}{\left|\left\langle v\left|{{\mathcal{H}}}^{{\prime} }\right|c\right\rangle \right|}^{2}\frac{2}{{\left(2\uppi \right)}^{3}}\delta \left({E}_{{{c}}}\left({\bf{k}}\right)-{E}_{{{v}}}\left({\bf{k}}\right)-{{\hslash }}\omega \right){{\mathrm{d}}}^{3}k,$$

where ${{\mathcal{H}}}^{{\prime} }$ is the perturbation associated with the light wave and $\left\langle v\left|{{\mathcal{H}}}^{{\prime} }\right|c\right\rangle$ is the transition matrix from states in the VB to states in the conduction band (CB); *δ* is the Dirac delta function that switches on this contribution when a transition is allowed, that is ${E}_{c}\left({\bf{k}}\right)-{E}_{v}\left({\bf{k}}\right)={{\hslash }}\omega$. The factor 2 stems from the spin degeneracy. The integration is over the entire Brillouin zone (BZ). The matrix elements vary little within the BZ. Therefore, we can pull these out in front of the integral and obtain

2 $$\frac{2\uppi }{\varOmega {{\hslash }}}{\left|\left\langle v\left|{{\mathcal{H}}}^{{\prime} }\right|c\right\rangle \right|}^{2}\int \frac{2\varOmega }{{\left(2\uppi \right)}^{3}}\delta \left({E}_{c}\left({\bf{k}}\right)-{E}_{v}\left({\bf{k}}\right)-{{\hslash }}\omega \right){\text{d}}^{3}k,$$

where *Ω* is the volume of the lattice cell, and the factor *Ω*/(2π)^3^ normalizes the **k** vector density within the BZ. The second term is the JDOS. After summing over all states within the first BZ and all possible transitions initiated by photons with a certain energy ${{\hslash }}\omega$ between the VB and CB, we obtain

3 $$\begin{array}{c}j\left(\omega \right)=\mathop{\sum }\limits_{v,c}\displaystyle\frac{\varOmega }{4{\uppi }^{3}}\int \delta \left({E}_{c}\left({\mathbf{k}}\right)-{E}_{v}\left({\mathbf{k}}\right)-{{\hslash }}\omega \right){{\mathrm{d}}}^{3}k\\ =2\mathop{\sum }\limits_{v,c,{\mathbf{k}}}{w}_{{\mathbf{k}}}\delta \left({E}_{c}\left({\mathbf{k}}\right)-{E}_{v}\left({\mathbf{k}}\right)-{{\hslash }}\omega \right)\end{array},$$

where *c* and *v* belong respectively to the VB and CB, *E*(**k**) are the eigenvalues of the Hamiltonian and *w*_**k**_ are weighting factors. The Dirac delta function in equation (3) can be numerically approximated by means of a normalized Gaussian function

4 $$G\left(\omega \right)=\frac{1}{\sigma \sqrt{2\uppi }}{{\mathrm{e}}}^{-{\left({E}_{{\mathbf{k}},n{\prime} }-{E}_{{\mathbf{k}},n}-{{\hslash }}\omega \right)}^{2}/2{\sigma }^{2}},$$

where *σ* is the broadening parameter.

## Online content

Any methods, additional references, Nature Portfolio reporting summaries, source data, extended data, supplementary information, acknowledgements, peer review information; details of author contributions and competing interests; and statements of data and code availability are available at 10.1038/s41563-025-02123-y.

## Supplementary information

Supplementary Information Supplementary Figs. 1–9, Notes 1 and 2 and Tables 1 and 2.

## Source data

Source Data Fig. 1 1b, J-V-L data of the PeLED; 1c, EQE-J data; 1d, statistical EQE_max_; 1e, *L*–*V* data of three different PeLEDs with different electron-transporting materials.

Source Data Fig. 2 2b, simulated JDOS results; 2c, experimental absorption results; 2d, UPS results of two samples; 2e, UPS results of one sample at two temperatures.

Source Data Fig. 3 3b, PL spectrum; 3c, PL lifetime; 3e, PL intensity versus PL lifetime.

Source Data Fig. 4 4a, simulated PL intensity versus carrier density; 4b, experimental PL intensity versus excitation power; 4c, normalized EQE_max_ at different temperatures.

Source Data Extended Data Fig. 1 TA results of perovskite thin film in both UV range and visible range.

Source Data Extended Data Fig. 2 UPS results of PMMA layer.

Source Data Extended Data Fig. 3 UPS results of PTAA layer.

Source Data Extended Data Fig. 4 UPS results of pristine perovskite thin film.

Source Data Extended Data Fig. 5 *J*–*V*–*L* results of PeLEDs with different device structures.

Source Data Extended Data Fig. 6 Simulated DOS results of perovskite layers with more (configuration A) and fewer (configuration B) additives.

Source Data Extended Data Fig. 7 XPS results of perovskite thin films before and after IPA wash.

Source Data Extended Data Fig. 8 UPS results of perovskite thin film after IPA wash.

Source Data Extended Data Fig. 10 PL spectrum and PL lifetime results of perovskite thin films with different numbers of additives.

## Data Availability

All data are available in the article or its Supplementary Information. Source data are provided with this paper.
