# Supplementary material for: Surfactant-induced hole concentration enhancement for highly efficient perovskite light-emitting diodes
Source: Nat Mater. 2025 Mar 5;24(5):778–84. doi: 10.1038/s41563-025-02123-y (PMC12048353; doi:10.1038/s41563-025-02123-y)
Supplement: Supplementary file 1 — Supplementary Figs. 1–9, Notes 1 and 2 and Tables 1 and 2. [file 41563_2025_2123_MOESM1_ESM.pdf]

nature materials

Supplementary information https://doi.org/10.1038/s41563-025-02123-y

Surfactant-induced hole concentration enhancement for highly efficient perovskite light-emitting diodes

In the format provided by the authors and unedited

Table of Contents

Note 1 Defect passivation in the *ABC* model. 2

Note 2 Simulation details about the doping induced QY enhancement. 2

Supplementary Fig. 1 | Absorption spectrum of our perovskite film. 3

Supplementary Fig. 2 | Device architecture of our PeLED. 4

Supplementary Fig. 3 | Optical bandgap extraction of our perovskite thin film. 5

Supplementary Fig. 4 | Current density-voltage (J-V) curves of the hole-only device. 6

Supplementary Fig. 5 | EL spectra of our PeLED at different biases. 7

Supplementary Fig. 6 | Schematic diagram on how to control the amount of additives and the corresponding absorption spectra. 8

Supplementary Fig. 7 | Conducting atomic force microscopy (C-AFM) results. 9

Supplementary Fig. 8 | Extension to 3D $MAPbBr_3$ perovskite systems. 10

Supplementary Fig. 9 | Extension to 3D $MAPbI_3$ perovskite systems. 11

Table 1 Summary of the hole transporting layers in Fig. 1f. 12

Table 2 Detailed stochiometric ratios of different perovskite solutions. 13

Supplementary References 13

Note 1 Defect passivation in the *ABC* model.

According to the *ABC* model, the change of generated carrier density n in metal halide perovskites can be expressed as [1]

$$-\frac{dn}{dt} = -G + an + bn^2 + cn^3 \quad (1)$$

where t is the time, G is the carrier generation rate, and a, b and c are first-order, second-order and third-order recombination rate coefficients, respectively. For perovskite systems with small exciton binding energy, only the second-order recombination contributes to the light emission and the first-order recombination contributes to the trap assisted nonradiative recombination loss. In the condition of characterizing the PL lifetime, high order terms (bn^2 and cn^3) are negligible at low carrier densities ($an \gg bn^2, cn^3$). Thus, the rate equation can be rewritten as

$$-\frac{dn}{dt} \approx an \quad (2)$$

Then we can obtain carrier density $n = n_0 e^{-at}$, and the radiative recombination rate $\mathbf{\Gamma_r} = bn^2 = bn_0^2 e^{-2at}$. Thus, the PL lifetime $\tau = \frac{1}{2a} \propto \frac{1}{a}$. Considering that $QY \approx \frac{bn^2}{an} \propto \frac{1}{a}$, the enhanced multiple of PL lifetime should be equal to the enhanced multiple of QY.

Note 2 Simulation details about the doping induced QY enhancement.

As the bimolecular recombination or free electron-hole recombination, the recombination rate is proportional to the product of electron density (n) and hole density (p), i.e. $\mathbf{\Gamma_r} = b\, n \times p$. In the p-type doped perovskites under photoexcitation (photogenerated electron density n_0 equals to the photogenerated hole density p_0), the total hole density $p = p_0 + p_D$, where p_D is the doping density. Thus, $\mathbf{\Gamma_r} = bn \times p = bn_0 \times (p_0 + p_D) = bn_0 \times (n_0 + p_D)$. In the simulation of Figure 4a, the PL intensity is proportional to $\mathbf{\Gamma_r}$.

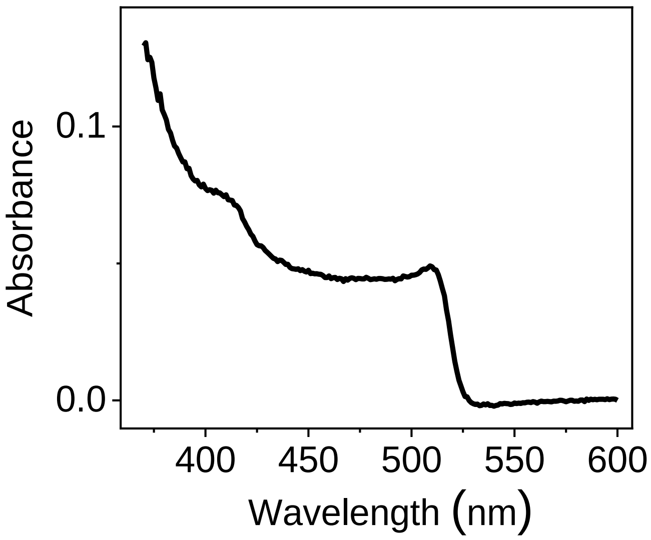

Supplementary Fig. 1 | Absorption spectrum of the perovskite film.

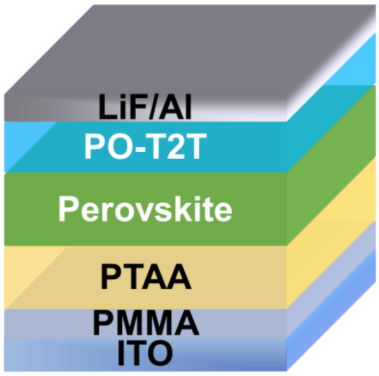

Supplementary Fig. 2 | Device architecture of the PeLED.

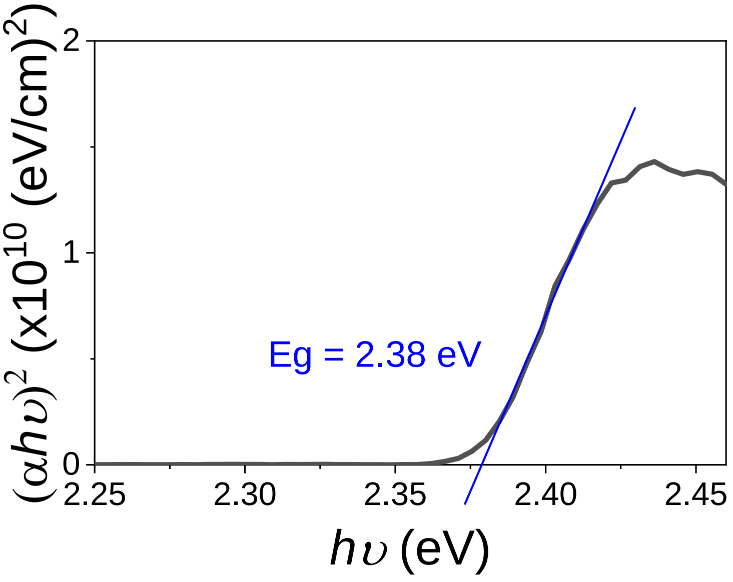

Supplementary Fig. 3 | Extraction of the optical bandgap of our perovskite thin film from the UV-vis absorption result.

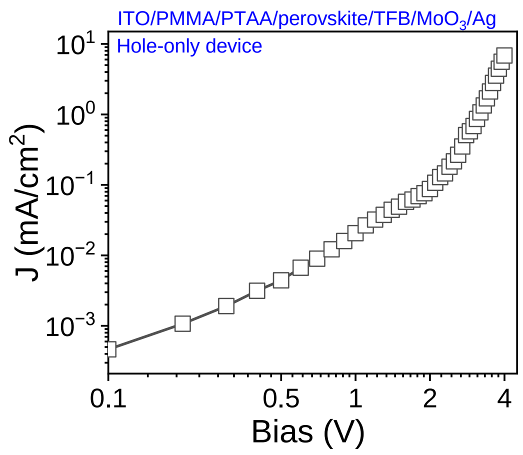

Supplementary Fig. 4 | Current density-voltage (J-V) curves of the hole-only device with an architecture of ITO/PMMA/PTAA/perovskite/TFB/MoO3/Ag.

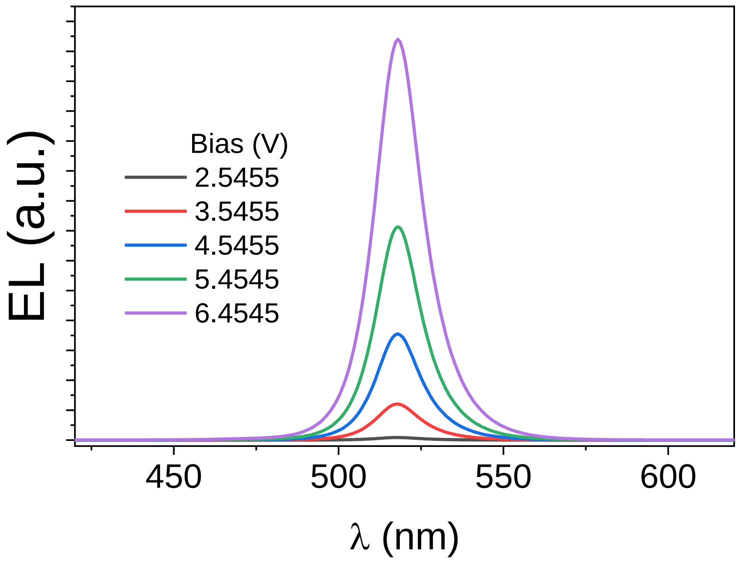

Supplementary Fig. 5 | EL spectra of our PeLED at different biases.

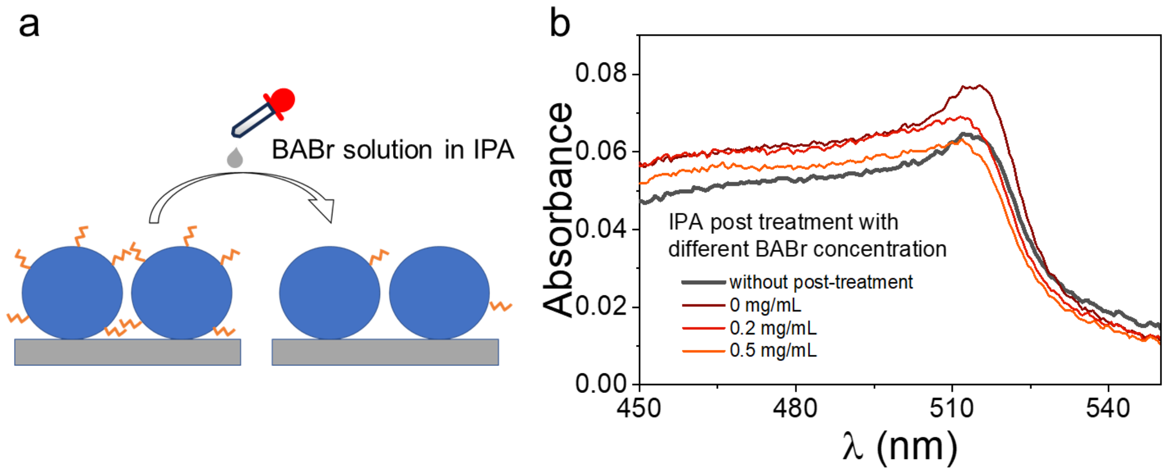

Supplementary Fig. 6 | a. Schematic diagram on how to control the amount of additives through changing the concentration of BABr solution in IPA. b. The corresponding absorption spectra with different BABr solution treatment, where 0 mg/ml means pure solvent wash.

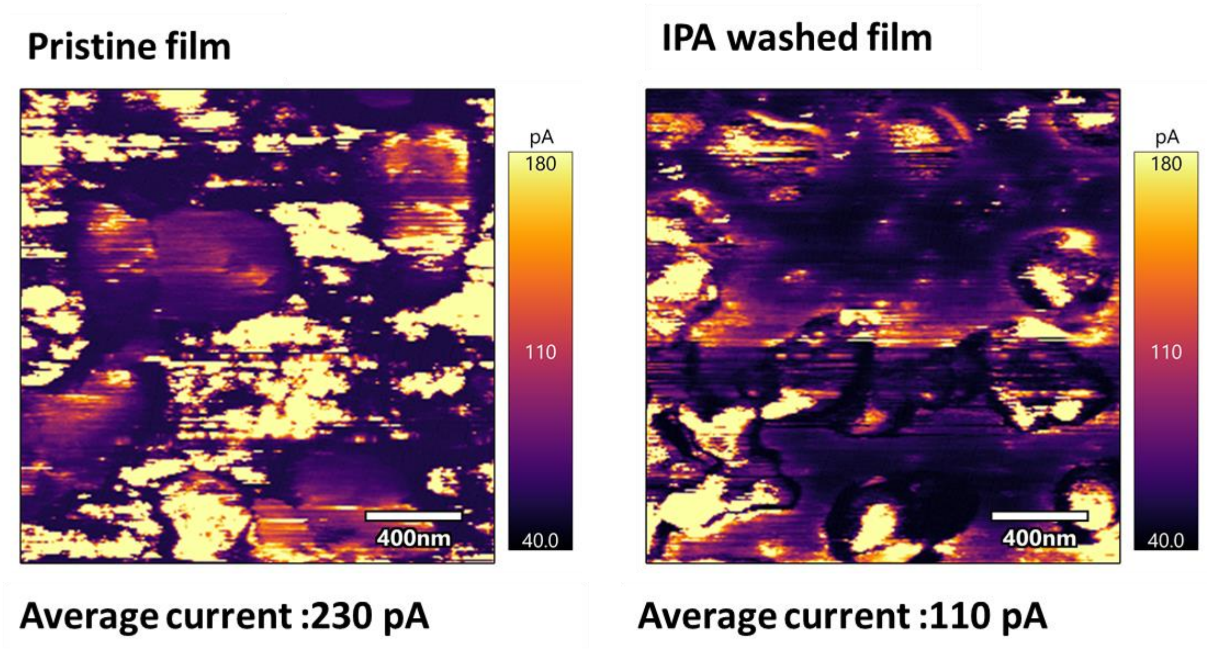

Supplementary Fig. 7 | Conducting atomic force microscopy (C-AFM) of our pristine perovskite film (left) and the perovskite film after IPA wash (right).

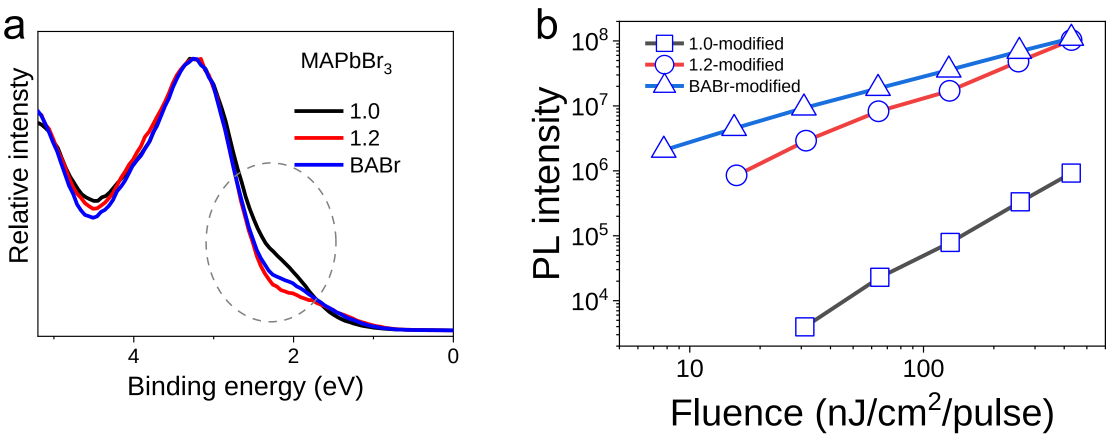

Supplementary Fig. 8 | Extension to 3D methylammonium lead bromide ($MAPbBr_3$) perovskite systems. a. UPS spectra and **b**. Fluence dependent PL intensity curves of 3 different $MAPbBr_3$ samples (1.0 refers to the reference sample with exactly the ideal stochiometric ratio for perovskite formation, 1.2 refers to the sample with addition of 20% excess MABr in the precursor, and BABr refers to the sample with addition of BABr additive in the precursor).

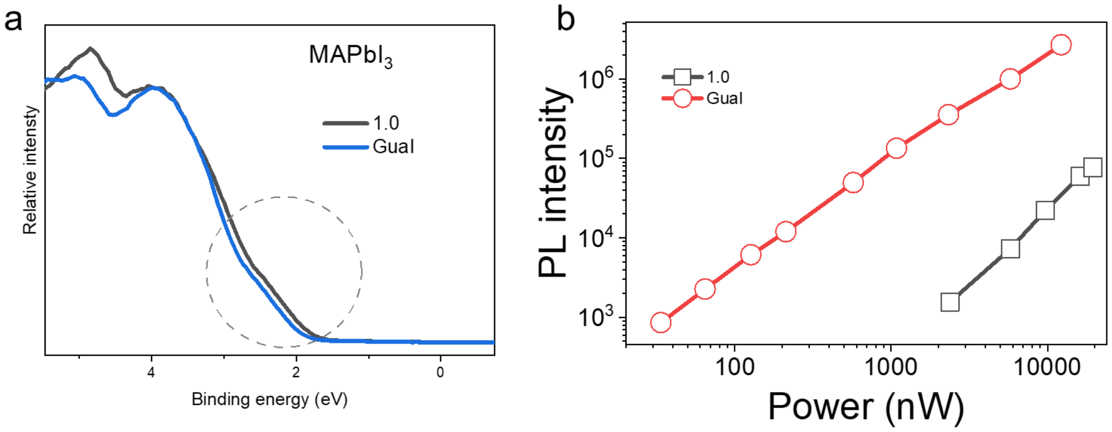

Supplementary Fig. 9 | Extension to 3D methylammonium lead iodide ($MAPbI_3$) perovskite systems. a. UPS spectra and **b**. Fluence dependent PL intensity curves of 2 different $MAPbI_3$ samples (1.0 refers to the reference sample with exactly the ideal stochiometric ratio for perovskite formation, and GuaI refers to the sample with addition of guanidinium iodide additive in the precursor).

Table 1 Summary of the hole transporting layers in Fig. 1f.

ETL	HTL(s)	Hole injection barrier(s)	HTL mobility ($cm^2\ V^{-1}\ s^{-1}$)	Turn-on voltage (V)	Ref.
TPBi	NiOx	0.46-0.80 eV	3.75×10^{-3} [2]	2.74-3.36	40,41
	TFB/LiF	~0.6 eV	10^{-2} [3]	3.0	44
	PVK	~0.4 eV	10^{-6} [3]	2.8	38
	PEDOT:PSS/poly-TPD	0.1+0.9 eV	$2x10^{-4} + 10^{-4}$ [4,5]	2.7	23
	PMMA/PTAA	/	Insulator + 10^{-3} [3]	2.6	our work
ZADN	PEDOT:PSS	NA	2×10^{-4} [3]	2.3	37
	PMMA/PTAA	/	Insulator + 10^{-3} [3]	2.2	our work
PO-T2T	TFB/LiF	~0.6 eV	10^{-2} [3]	2.0	43
	PMMA/PTAA	/	Insulator + 10^{-3} [3]	2.0	our work
ZnO	CBP/TFB	0.8+0 eV	$2\times10^{-3} + 10^{-2}$ [3,6]	1.85	42
	MoO3/poly-TPD	0.4 eV	10^{-4} [5]	1.7	39

Table 2 Detailed stochiometric ratios of different perovskite solutions.

<table>
<tr><th rowspan="2"></th><th colspan="4">Solution A in 3 mL DMSO (200 μL)</th><th rowspan="2">SPPO13 in DMSO (200 μL)</th><th rowspan="7">EA
(1 μL)</th></tr>
<tr><th>BABr</th><th>CsBr</th><th>PbBr2</th><th>PEO</th></tr>
<tr><td>**1**</td><td>0.0393g</td><td rowspan="5">0.1277g</td><td rowspan="5">0.2202g</td><td>0.025g</td><td>0</td></tr>
<tr><td>**2**</td><td>0.0416g</td><td>0.025g</td><td>0</td></tr>
<tr><td>**3**</td><td>0.0439g</td><td>0.050g</td><td>0</td></tr>
<tr><td>**4**</td><td>0.0439g</td><td>0.075g</td><td>0</td></tr>
<tr><td>**5**</td><td>0.0439g</td><td>0.075g</td><td>8 mg/mL</td></tr>
</table>

Supplementary References

1. Qin, J., Liu, X.-K., Yin, C. & Gao, F. Carrier dynamics and evaluation of lasing actions in halide perovskites. *Trends Chem.* **3**, 34–46 (2021).
2. Jiang, F., Choy, W. C. H., Li, X. C., Zhang, D. & Cheng, J. Q. Post-treatment-free solution-processed non-stoichiometric niox nanoparticles for efficient hole-transport layers of organic optoelectronic devices. *Adv. Mater.* **27**, 2930–2937 (2015).
3. Lu, Y. et al. Tuning Hole Transport Layers and Optimizing Perovskite Films Thickness for High Efficiency $CsPbBr_3$ Nanocrystals Electroluminescence Light-Emitting Diodes. *J. Lumin.* **234**, 117952 (2021).
4. Huang, Q. et al. High-performance quantum dot light-emitting diodes with hybrid hole transport layer via doping engineering. *Opt. Express* **24**, 25955– 25963 (2016).
5. Wang, Y. et al. High-Performance Hole Transport Layer Based on WS2 Doped PEDOT:PSS for Organic Solar Cells. *Organic Electronics* **99**, 106305 (2021).
6. Xiang, C., Koo, W., So, F., Sasabe, H. & Kido, J. A systematic study on efficiency enhancements in phosphorescent green, red and blue microcavity organic light emitting devices. *Light Sci. Appl.* **2**, e74 (2013).
